# RETRACTION: PRMT5 Participates in B Cell Overactivation in Patients With Primary Sjogren's Syndrome (pSS) Through RSAD2‐Mediated NF‐κB Signaling

**DOI:** 10.1002/iid3.70269

**Published:** 2025-09-29

## Abstract

**RETRACTION:** H. Zhu, J. Zheng, Y. Zhou, T. Wu, and T. Zhu, “PRMT5 Participates in B Cell Overactivation in Patients With Primary Sjogren's Syndrome (pSS) Through RSAD2‐Mediated NF‐κB Signaling,” *Immunity, Inflammation and Disease* 11, no. 12 (2023): e1102, https://doi.org/10.1002/iid3.1102.

The above article, published online on 13 December 2023 in Wiley Online Library (wileyonlinelibrary.com), has been retracted by agreement between the authors; the journal Editor‐in‐Chief, Marc Veldhoen; and John Wiley & Sons, Ltd. The retraction has been agreed upon due to the identification of duplication of the p‐p65 western blot bands between Figures 4C and 6E. The duplication consists of rotated versions of the same bands, which were used to represent different scientific conditions. Discrepancies were also noted in the participant profile and funding information. The study included an unusually high proportion of male participants for a condition known to predominantly affect women. Additionally, the funding statement lacked institutional attribution and appeared misaligned with the study's focus. The authors did not respond to invitations to comment on the concerns or provide supporting data. As a result, the editors consider the results and conclusions to be compromised.

**RETRACTION:** H. Zhu, J. Zheng, Y. Zhou, T. Wu, and T. Zhu, “PRMT5 Participates in B Cell Overactivation in Patients With Primary Sjogren's Syndrome (pSS) Through RSAD2‐Mediated NF‐κB Signaling,” *Immunity, Inflammation and Disease* 11, no. 12 (2023): e1102, https://doi.org/10.1002/iid3.1102.

The above article, published online on 13 December 2023 in Wiley Online Library (wileyonlinelibrary.com), has been retracted by agreement between the authors; the journal Editor‐in‐Chief, Marc Veldhoen; and John Wiley & Sons, Ltd. The retraction has been agreed upon due to the identification of duplication of the p‐p65 western blot bands between Figures 4C and 6E. The duplication consists of rotated versions of the same bands, which were used to represent different scientific conditions. Discrepancies were also noted in the participant profile and funding information. The study included an unusually high proportion of male participants for a condition known to predominantly affect women. Additionally, the funding statement lacked institutional attribution and appeared misaligned with the study's focus. The authors did not respond to invitations to comment on the concerns or provide supporting data. As a result, the editors consider the results and conclusions to be compromised.

